# Supplementary material for: Morphological and Phylogenetic Analyses Reveal Three New Species of Pestalotiopsis (Sporocadaceae, Amphisphaeriales) from Hainan, China
Source: Microorganisms. 2023 Jun 21;11(7):1627. doi: 10.3390/microorganisms11071627 (PMC10385101; doi:10.3390/microorganisms11071627)
Supplement: Supplementary file 1 [file microorganisms-11-01627-s001.zip › Table S1.pdf]

Table S1. Information of specimens used in this study.

Fungal species	Voucher	Substrate	Country	GenBank accession		
				ITS	*tef1α*	*tub2*
Neopestalotiopsis magna	MFLUCC 12-0652*	*Pteridium* sp.	France	KF582795	KF582791	KF582793
Pestalotiopsis abietis	CFCC 53011*	*Abies fargesii*	China	MK397013	MK622277	MK622280
	CFCC 53012	*Abies fargesii*	China	MK397014	MK622278	MK622281
	CFCC 53013	*Abies fargesii*	China	MK397015	MK622279	MK622282
P. adusta	ICMP 6088*	Refrigerator door	Fiji	JX399006	JX399070	JX399037
	MFLUCC 10-146	*Syzygium* sp.	Thailand	JX399007	JX399071	JX399038
P. aggestorum	LC6301*	*Camellia sinensis*	China	KX895015	KX895234	KX895348
	LC8186	*Camellia sinensis*	China	KY464140	KY464150	KY464160
P. anacardiacearum	IFRDCC 2397*	*Mangifera indica*	China	KC247154	KC247156	KC247155
P. anhuiensis	CFCC 54791*	*Cyclobalanopsis glauca*	China	ON007028	ON005045	ON005056
P. arceuthobii	CBS 434.65*	*Arceuthobium campylopodum*	USA	KM199341	KM199516	KM199427
P. arengae	CBS 331.92*	*Arenga undulatifolia*	Singapore	KM199340	KM199515	KM199426
P. australasiae	CBS 114126*	*Knightia* sp.	New Zealand	KM199297	KM199499	KM199409
	CBS 114141	*Protea* sp.	New South Wales	KM199298	KM199501	KM199410
P. australis	CBS 111503	*Protea neriifolia*	South Africa	KM199331	KM199557	KM199382
	CBS 114193*	*Grevillea* sp.	New South Wales	KM199332	KM199475	KM199383
P. biciliata	CBS 124463*	*Platanus hispanica*	Slovakia	KM199308	KM199505	KM199399
	CBS 236.38	*Paeonia* sp.	Italy	KM199309	KM199506	KM199401
P. brachiata	LC2988*	*Camellia* sp.	China	KX894933	KX895150	KX895265
	LC8188	*Camellia* sp.	China	KY464142	KY464152	KY464162
	LC8189	*Camellia* sp.	China	KY464143	KY464153	KY464163
P. brassicae	CBS 170.26*	*Brassica napus*	New Zealand	KM199379	KM199558	-
P. camelliae	MFLUCC 12-0277*	*Camellia japonica*	China	JX399010	JX399074	JX399041
P. camelliae-oleiferae	CSUFTCC08*	*Camelliae oleiferae*	China	OK493593	OK507963	OK562368
	CSUFTCC09	*Camelliae oleiferae*	China	OK493594	OK507964	OK562369
P. castanopsidis	CFCC 54430*	*Castanopsis lamontii*	China	OK339732	OK358493	OK358508
	CFCC 54305	*Castanopsis hystrix*	China	OK339733	OK358494	OK358509
	CFCC 54384	*Castanopsis hystrix*	China	OK339734	OK358495	OK358510
P. chamaeropis	CBS 186.71*	*Chamaerops humilis*	Italy	KM199326	KM199473	KM199391
	LC3619	*Camellia* sp.	China	KX894991	KX895208	KX895322
	CFCC 55124	*Quercus acutissima*	China	OM746221	OM839993	OM839894
	CFCC 55019	*Quercus aliena*	China	OM746224	OM839996	OM839897
	CFCC 55023	*Castanopsis fissa*	China	OM746233	OM840005	OM839906
	CFCC 54977	*Quercus acutissima*	China	OM746223	OM839995	OM839896
	CFCC 55122	*Quercus aliena*	China	OM746229	OM840001	OM839902
	CFCC 54776	*Quercus variabilis*	China	OM746234	OM840006	OM839907
P. changjiangensis	CFCC 54314*	*Castanopsis tonkinensis*	China	OK339739	OK358500	OK358515
	CFCC 54433	*Castanopsis hainanensis*	China	OK339740	OK358501	OK358516
	CFCC 52803	*Cyclobalanopsis* sp.	China	OK339741	OK358502	OK358517
P. chiaroscuro	BRIP 72970*	*Sporobolus natalensis*	Australia	OK422510	OK423753	OK423752
P. chinensis	MFLUCC 12-0273	*Taxus* sp.	China	JX398995	-	-
P. clavata	MFLUCC 12-0268*	*Buxus* sp.	China	JX398990	JX399056	JX399025
P. colombiensis	CBS 118553*	*Eucalyptus urograndis*	Colombia	KM199307	KM199488	KM199421
P. cyclobalanopsidis	CFCC 54328*	*Cyclobalanopsis glauca*	China	OK339735	OK358496	OK358511
	CFCC 55891	*Cyclobalanopsis glauca*	China	OK339736	OK358497	OK358512
P. dianellae	CPC 32261	*Dianella* sp.	Australia	MG386051	-	MG386164

P. digitalis	MFLU 14-0208*	*Digitalis purpurea*	New Zealand	KP781879	-	KP781883
P. dilucida	LC3232*	*Camellia sinensis*	China	KX894961	KX895178	KX895293
	LC8184	*Camellia sinensis*	China	KY464138	KY464148	KY464158
P. diploclisiae	CBS 115449	*Psychotria tutcheri*	China	KM199314	KM199485	KM199416
	CBS 115587*	*Diploclisia glaucescens*	China	KM199320	KM199486	KM199419
P. disseminata	CBS 143904	*Persea americana*	New Zealand	MH554152	MH554587	MH554825
	MEAN 1165	*Pinus pinea*	Portugal	MT374687	MT374699	MT374712
P. diversiseta	MFLUCC 12-0287*	*Rhododendron* sp.	China	JX399009	JX399073	JX399040
P. doitungensis	MFLUCC 14-0115*	*Dendrobium* sp.	Thailand	MK993574	MK975832	MK975837
P. dracaenicola	MFLUCC 18-0913*	*Dracaena* sp.	Thailand	MN962731	MN962732	MN962733
P. dracontomelonis	MFLU 14-0207*	*Dracontomelon* sp.	Thailand	KP781877	KP781880	-
P. endophytica	MFLU 20-0607*	*Magnolia garrettii*	Thailand	MW263946	MW417119	-
P. ericacearum	IFRDCC 2439*	*Rhododendron delavayi*	China	KC537807	KC537814	KC537821
P. etonensis	BRIP 66615*	*Sporobolus jacquemontii*	Australia	MK966339	MK977635	MK977634
P. ficicola	**SAUCC230046***	***Ficus microcarpa***	**China**	**OQ691974**	**OQ718691**	**OQ718749**
	**SAUCC230042**	***Ficus microcarpa***	**China**	**OQ691972**	**OQ718689**	**OQ718747**
	**SAUCC230043**	***Ficus microcarpa***	**China**	**OQ691973**	**OQ718690**	**OQ718748**
P. foliicola	CFCC 54440*	*Castanopsis faberi*	China	ON007029	ON005046	ON005057
	CFCC 57359	*Castanopsis faberi*	China	ON007030	ON005047	ON005058
	CFCC 57360	*Castanopsis faberi*	China	ON007031	ON005048	ON005059
P. formosana	NTUCC 17-009*	*Poaceae* sp.	China	MH809381	MH809389	MH809385
P. furcata	MFLUCC 12-0054*	*Camellia sinensis*	Thailand	JQ683724	JQ683740	JQ683708
	LC6691	*Camellia sinensis*	China	KX895030	KX895248	KX895363
P. gaultheriae	IFRD 411-014*	*Gaultheria forrestii*	China	KC537805	KC537812	KC537819
P. gibbosa	NOF 3175*	*Gaultheria shallon*	Canada	LC311589	LC311591	LC311590
P. grandis-urophylla	E-72-02	*Eucalyptus* sp.	Brazil	KU926708	KU926712	KU926716
	E-72-03	*Eucalyptus* sp.	Brazil	KU926709	KU926713	KU926717
	E-72-04	*Eucalyptus* sp.	Brazil	KU926710	KU926714	KU926718
	E-72-06	*Eucalyptus* sp.	Brazil	KU926711	KU926715	KU926719
P. grevilleae	CBS 114127*	*Grevillea* sp.	Australia	KM199300	KM199504	KM199407
P. guangxiensis	CFCC 54308*	*Quercus griffithii*	China	OK339737	OK358498	OK358513
	CFCC 54300	*Quercus griffithii*	China	OK339738	OK358499	OK358514
P. guizhouensis	CFCC 54803	*Cyclobalanopsis glauca*	China	ON007035	ON005052	ON005063
	CFCC 57364	*Cyclobalanopsis glauca*	China	ON007036	ON005053	ON005064
P. hawaiiensis	CBS 114491*	*Leucospermum* sp.	USA	KM199339	KM199514	KM199428
P. hispanica	CBS 115391*	*Protea* sp.	Spain	MH553981	MH554399	MH554640
P. hollandica	CBS 265.33*	*Sciadopitys verticillata*	The Netherlands	KM199328	KM199481	KM199388
P. humicola	CBS 336.97*	Soil	Papua New Guinea	KM199317	KM199484	KM199420
P. hunanensis	CSUFTCC15*	*Camellia oleifera*	China	OK493599	OK507969	OK562374
P. hunanensis	CSUFTCC18	*Camellia oleifera*	China	OK493600	OK507970	OK562375
P. hydei	MFLUCC 20-0135	*Litsea elliptica*	Thailand	MW266063	MW251113	MW251112
P. iberica	CAA 1004*	*Pinus radiata*	Spain	MW732248	MW759038	MW759035
	CAA 1005	*Pinus sylvestris*	Spain	MW732250	MW759037	MW759034
	CAA 1006	*Pinus radiata*	Spain	MW732249	MW759039	MW759036
P. inflexa	MFLUCC 12-0270*	Unidentified tree	China	JX399008	JX399072	JX399039
P. intermedia	MFLUCC 12-0259*	Unidentified tree	China	JX398993	JX399059	JX399028
P. italiana	MFLU 14-0214*	*Cupressus glabra*	Italy	KP781878	KP781881	KP781882
P. jesteri	CBS 109350*	*Fragraea bodenii*	Papua New Guinea	KM199380	KM199554	-
P. jiangxiensis	LC4399*	*Camellia* sp.	China	KX895009	KX895227	KX895341
P. jinchanghensis	LC6636*	*Camellia sinensis*	China	KX895028	KX895247	KX895361
	LC8190	*Camellia sinensis*	China	KY464144	KY464154	KY464164

P. kaki	KNU-PT-1804*	*Diospyros kaki*	Korea	LC552953	LC553555	LC552954
P. kandelicola	NCYUCC 19-0354	*Kandelia candel*	China	MT560723	MT563102	MT563100
	NCYUCC 19-0355*	*Kandelia candel*	China	MT560722	MT563101	MT563099
P. kenyana	CBS 442.67*	*Coffea* sp.	Kenya	KM199302	KM199502	KM199395
	LC6633	*Camellia sinensis*	China	KX895027	KX895246	KX895360
P. knightiae	CBS 111963	*Knightia* sp.	New Zealand	KM199311	KM199495	KM199406
	CBS 114138*	*Knightia* sp.	New Zealand	KM199310	KM199497	KM199408
P. krabiensis	MFLUCC 16-0260*	*Pandanus* sp.	Thailand	MH388360	MH388395	MH412722
P. leucadendri	CBS 121417*	*Leucadendron* sp.	South Africa	MH553987	MH554412	MH554654
P. licualicola	HGUP 4057*	*Licuala grandis*	China	KC492509	KC481684	KC481683
	SAUCC210087	*Ilex chinensis*	China	OK087323	OK104879	OK104872
	SAUCC210088	*Ilex chinensis*	China	OK087324	OK104880	OK104873
P. lijiangensis	CFCC 50738*	*Castanopsis carlesii*	China	KU860520	KU844185	-
P. linearis	MFLUCC 12-0271*	*Trachelospermum* sp.	China	JX398992	JX399058	JX399027
P. lithocarpi	CFCC 55100*	*Lithocarpus chiungchungensis*	China	OK339742	OK358503	OK358518
	CFCC 55893	*Lithocarpus chiungchungensis*	China	OK339743	OK358504	OK358519
P. lushanensis	LC4344*	*Camellia* sp.	China	KX895005	KX895223	KX895337
	LC8182	*Camellia* sp.	China	KY464136	KY464146	KY464156
	LC8183	*Camellia* sp.	China	KY464137	KY464147	KY464157
	CFCC 54894	*Quercus serrata*	China	OM746282	OM840054	OM839955
P. macadamiae	BRIP 63738b	*Macadamia integrifolia*	Australia	KX186588	KX186621	KX186680
	BRIP 63739b	*Macadamia integrifolia*	Australia	KX186587	KX186620	KX186679
	BRIP 63741a	*Macadamia integrifolia*	Australia	KX186586	KX186619	KX186678
P. malayana	CBS 102220*	*Macaranga triloba*	Malaysia	KM199306	KM199482	KM199411
P. microspora	SS1-033I	*Cornus canadensis*	Canada	MT644300	-	-
P. monochaeta	CBS 144.97*	*Quercus robur*	The Netherlands	KM199327	KM199479	KM199386
	CBS 440.83	*Taxus baccata*	The Netherlands	KM199329	KM199480	KM199387
P. montellica	MFLUCC 12-0279*	dead plant material	China	JX399012	JX399076	JX399043
P. nanjingensis	CFCC 53882	*Quercus aliena*	China	OM746295	OM840067	OM839968
	CSUFTCC16*	*Camellia oleifera*	China	OK493602	OK507972	OK562377
P. nanningensis	CSUFTCC10*	*Camellia oleifera*	China	OK493596	OK507966	OK562371
P. neolitseae	NTUCC 17-011*	*Neolitsea villosa*	China	MH809383	MH809391	MH809387
	CFCC 54590	*Lithocarpus amygdalifolius*	China	OK339744	OK358505	OK358520
P. novae-hollandiae	CBS 130973*	*Banksia grandis*	Australia	KM199337	KM199511	KM199425
P. oryzae	CBS 111522	*Telopea* sp.	USA	KM199294	KM199493	KM199394
	CBS 171.26	Unknown host	Italy	KM199304	KM199494	KM199397
	CBS 353.69*	*Oryza sativa*	Denmark	KM199299	KM199496	KM199398
P. pallidotheae	MAFF 240993*	*Pieris japonica*	Japan	AB482220	-	-
P. pandanicola	MFLUCC 16-0255*	*Pandanus* sp.	Thailand	MH388361	MH388396	MH412723
P. papuana	CBS 331.96*	Coastal soil	Papua New Guinea	KM199321	KM199491	KM199413
	CBS 887.96	*Cocos nucifera*	Papua New Guinea	KM199318	KM199492	KM199415
P. parva	CBS 265.37	*Delonix regia*	-	KM199312	KM199508	KM199404
	CBS 278.35*	*Delonix regia*	-	KM199313	KM199509	KM199405
P. phoebes	**SAUCC230093***	***Phoebe zhennan***	**China**	**OQ692028**	**OQ718745**	**OQ718803**
	**SAUCC230092**	***Phoebe zhennan***	**China**	**OQ692027**	**OQ718744**	**OQ718802**
	**SAUCC230094**	***Phoebe zhennan***	**China**	**OQ692029**	**OQ718746**	**OQ718804**
P. photiniicola	YB28-2	Unknown	China	MK228997	MK512491	MK360938
P. pini	MEAN 1092	*Pinus pinea*	Portugal	MT374680	MT374693	MT374705

P. pinicola	KUMCC 19-0183*	*Pinus armandii*	China	MN412636	MN417509	MN417507
P. portugalica	CBS 393.48*	Unknown	Portugal	KM199335	KM199510	KM199422
	LC4324	*Camellia chekiangoleosa*	China	KX895001	KX895219	KX895333
P. rhizophorae	MFLUCC 17-0416*	*Rhizophora mucronata*	Thailand	MK764283	MK764327	MK764349
P. rhododendri	IFRDCC 2399*	*Rhododendron sinogrande*	China	KC537804	KC537811	KC537818
P. rhodomyrtus	CFCC 54733	*Quercus aliena*	China	OM746310	OM840082	OM839983
	CFCC 55052	*Cyclobalanopsis augustinii*	China	OM746311	OM840083	OM839984
P. rosea	MFLUCC 12-0258*	*Pinus* sp.	China	JX399005	JX399069	JX399036
P. scoparia	CBS 176.25*	*Chamaecyparis* sp.	China	KM199330	KM199478	KM199393
P. sequoiae	MFLUCC 13-0399*	*Sequoia sempervirens*	Italy	KX572339	-	-
P. shaanxiensis	CFCC 54958*	*Quercus variabilis*	China	ON007026	ON005043	ON005054
	CFCC 57356	*Quercus variabilis*	China	ON007027	ON005044	ON005055
P. shorea	MFLUCC 12-0314*	*Shorea obtusa*	Thailand	KJ503811	KJ503817	KJ503814
P. silvicola	CFCC 55296*	*Cyclobalanopsis kerrii*	China	ON007032	ON005049	ON005060
	CFCC 54915	*Cyclobalanopsis kerrii*	China	ON007033	ON005050	ON005061
	CFCC 57363	*Cyclobalanopsis kerrii*	China	ON007034	ON005051	ON005062
P. spatholobi	**SAUCC231201***	***Spatholobus suberectus***	**China**	**OQ692023**	**OQ718740**	**OQ718798**
	**SAUCC231203**	***Spatholobus suberectus***	**China**	**OQ692024**	**OQ718741**	**OQ718799**
	**SAUCC231204**	***Spatholobus suberectus***	**China**	**OQ692025**	**OQ718742**	**OQ718800**
	**SAUCC231213**	***Spatholobus suberectus***	**China**	**OQ692026**	**OQ718743**	**OQ718801**
P. spathulata	CBS 356.86*	*Gevuina avellana*	Chile	KM199338	KM199513	KM199423
P. spathuliappendiculata	CBS 144035*	*Phoenix canariensis*	Australia	MH554172	MH554607	MH554845
P. telopeae	CBS 114137	*Protea* sp.	Australia	KM199301	KM199559	KM199469
	CBS 114161*	*Telopea* sp.	Australia	KM199296	KM199500	KM199403
	CBS 113606	*Telopea* sp.	Australia	KM199295	KM199498	KM199402
P. terricola	CBS 141.69*	Soil	Pacific islands	MH554004	MH554438	MH554680
P. thailandica	MFLUCC 17-1616*	*Rhizophora mucronata*	Thailand	MK764285	MK764329	MK764351
P. trachycarpicola	OP068*	*Trachycarpus fortunei*	China	JQ845947	JQ845946	JQ845945
	IFRDCC 2403	*Podocarpus macrophyllus*	China	KC537809	KC537816	KC537823
	LC4523	*Camellia sinensis*	China	KX895011	KX895230	KX895344
P. unicolor	MFLUCC 12-0276*	*Rhododendron* sp.	China	JX398999	-	JX399030
	MFLUCC 12-0275	Unidentified tree	China	JX398998	JX399063	JX399029
P. verruculosa	MFLUCC 12-0274*	*Rhododendron* sp.	China	JX398996	JX399061	-
P. yanglingensis	LC4553*	*Camellia sinensis*	China	KX895012	KX895231	KX895345
	LC3412	*Camellia sinensis*	China	KX894980	KX895197	KX895312
P. yunnanensis	HMAS 96359*	*Podocarpus macrophyllus*	China	AY373375	-	-

Notes: Ex-type strains are marked with "*". Novel species introduced are in bold in this study.
